# Supplementary figures and images for: Ubiquitination of VE-cadherin regulates inflammation-induced vascular permeability in vivo
Source: EMBO Rep. 2024 Aug 7;25(9):17. doi: 10.1038/s44319-024-00221-7 (PMC11387630; doi:10.1038/s44319-024-00221-7)

# Figure 1B Blots

B

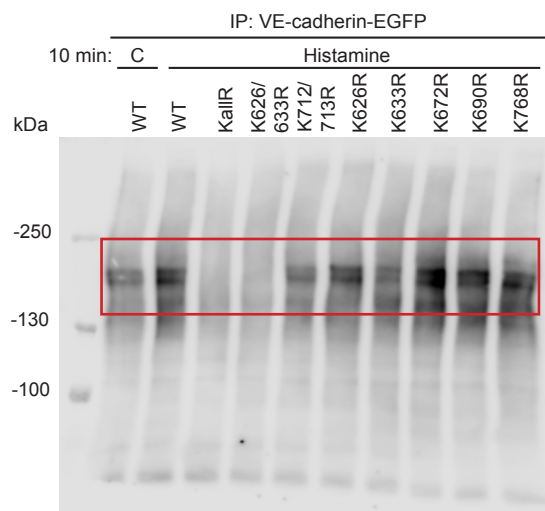

Ubiquitin

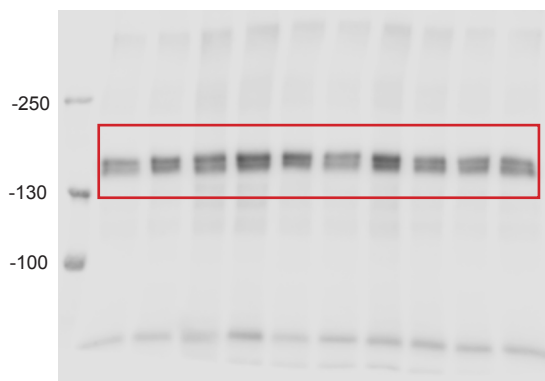

VE-cadherin-EGFP

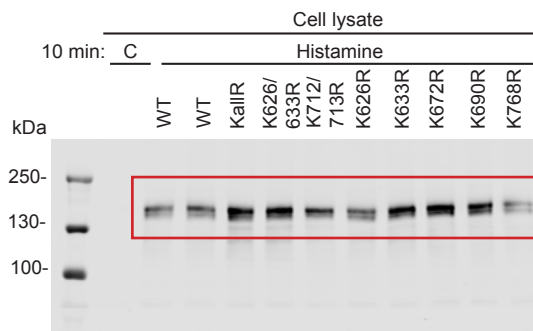

VE-cadherin-EGFP

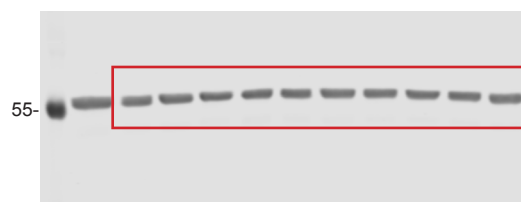

$\alpha$ -tubulin

Supplement: Supplementary file 2 — Source data Fig. 1 [file 44319_2024_221_MOESM2_ESM.zip › EMBOR-2023-58528V1_SourceDataForFigure1/1B/Figure_1B_Blots.pdf]

# Figure 1D Blots

D

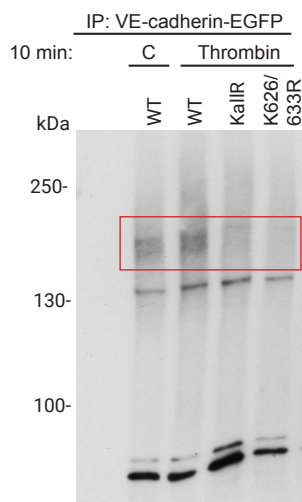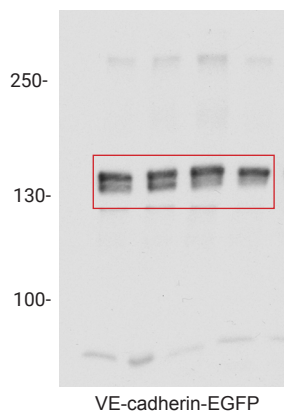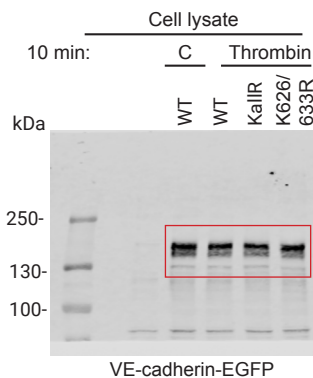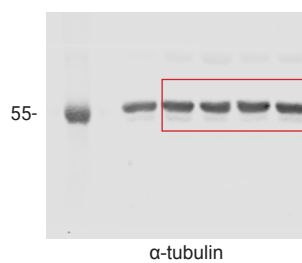

Supplement: Supplementary file 2 — Source data Fig. 1 [file 44319_2024_221_MOESM2_ESM.zip › EMBOR-2023-58528V1_SourceDataForFigure1/1D/Figure_1D_Blots.pdf]

# Figure 3E Blots

E

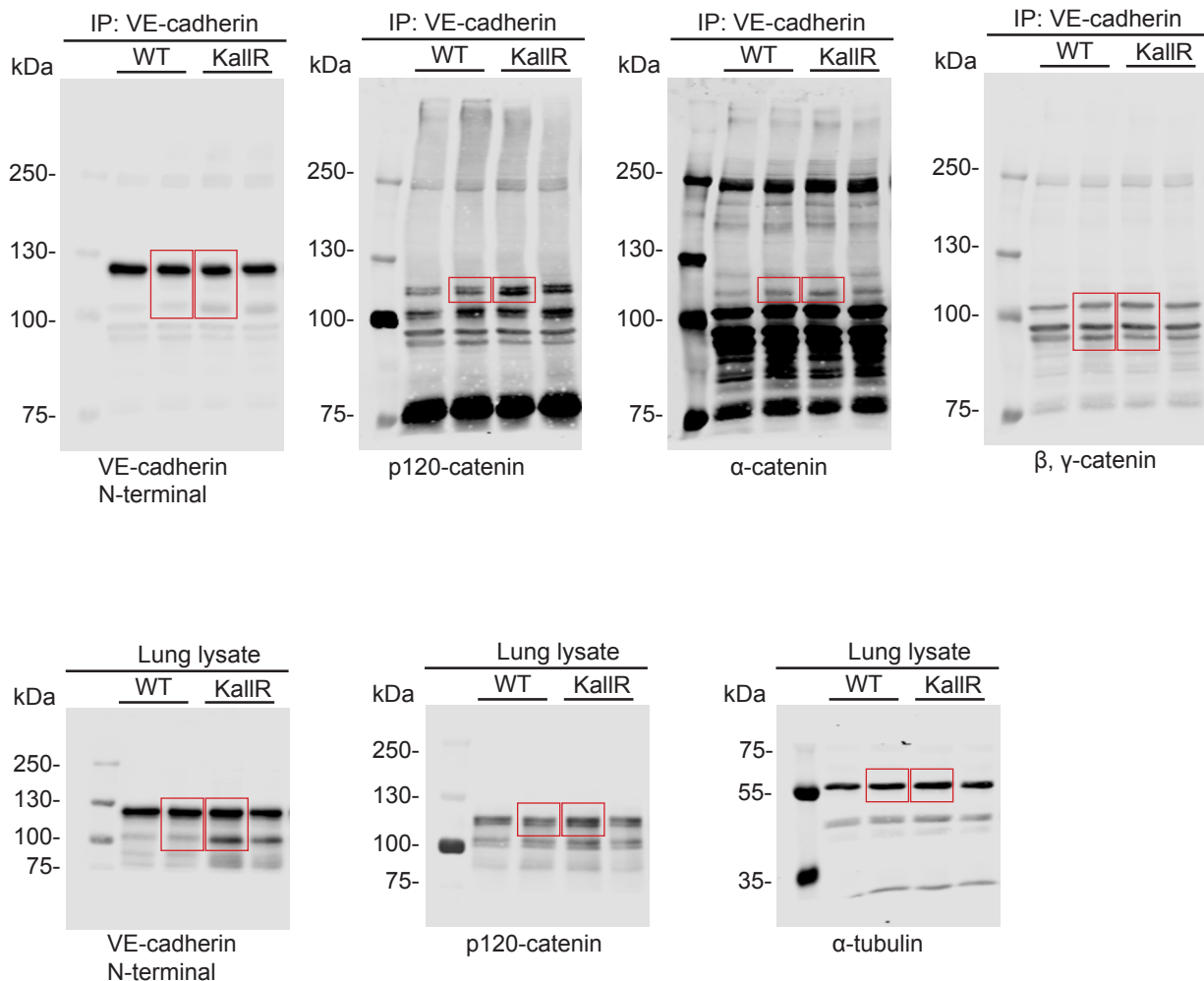

Blot of lung lysate was cut in half at 75 kDa

Supplement: Supplementary file 4 — Source data Fig. 3 [file 44319_2024_221_MOESM4_ESM.zip › EMBOR-2023-58528V1_SourceDataForFigure3/3E/Figure_3E_Blots.pdf]

# Figure 3H Blots

H

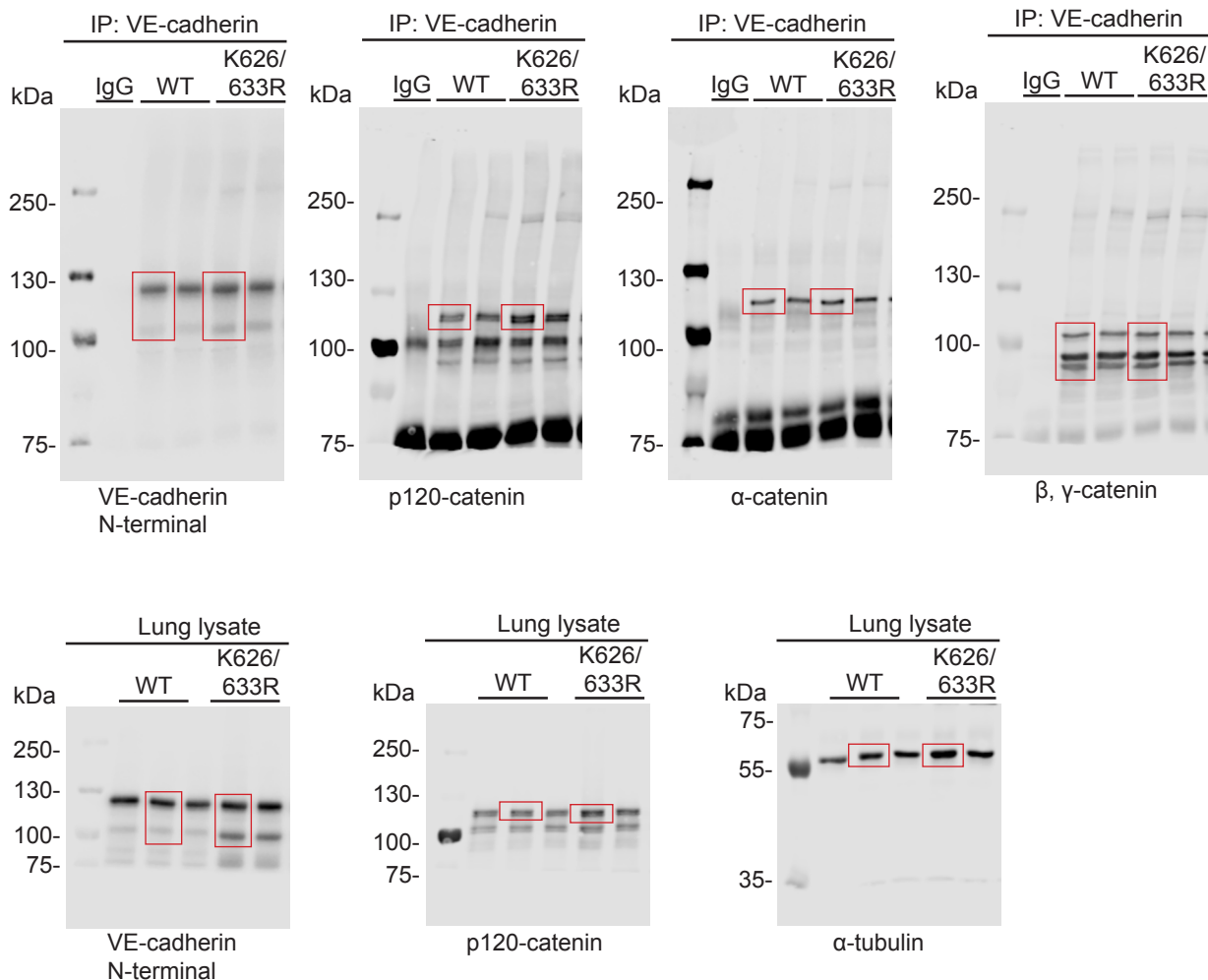

Blot of lung lysate was cut in half at 75 kDa

Supplement: Supplementary file 4 — Source data Fig. 3 [file 44319_2024_221_MOESM4_ESM.zip › EMBOR-2023-58528V1_SourceDataForFigure3/3H/Figure_3H_Blots.pdf]

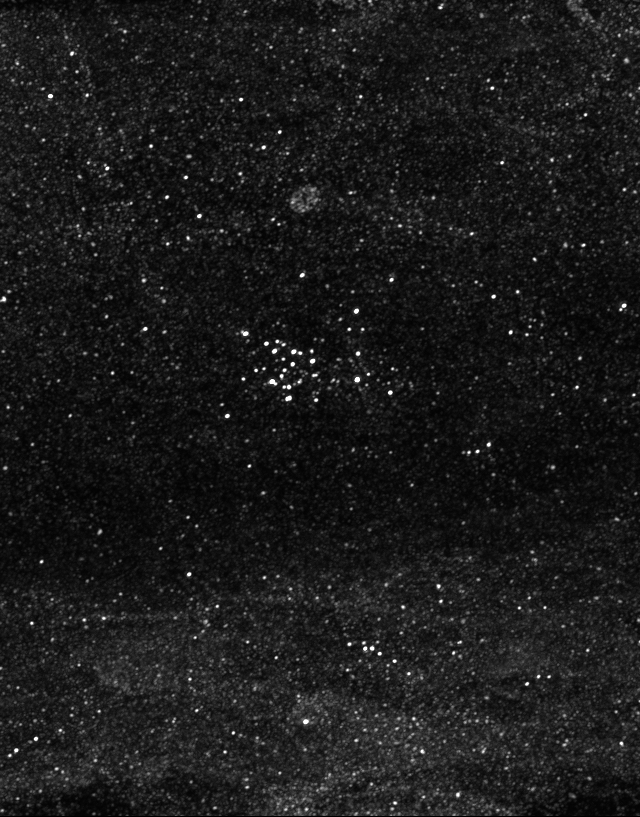

Supplement: Supplementary file 8 — Source data Fig. 7 [file 44319_2024_221_MOESM8_ESM.zip › EMBOR-2023-58528V1_SourceDataForFigure7/7B/KallR_MVB.tif]

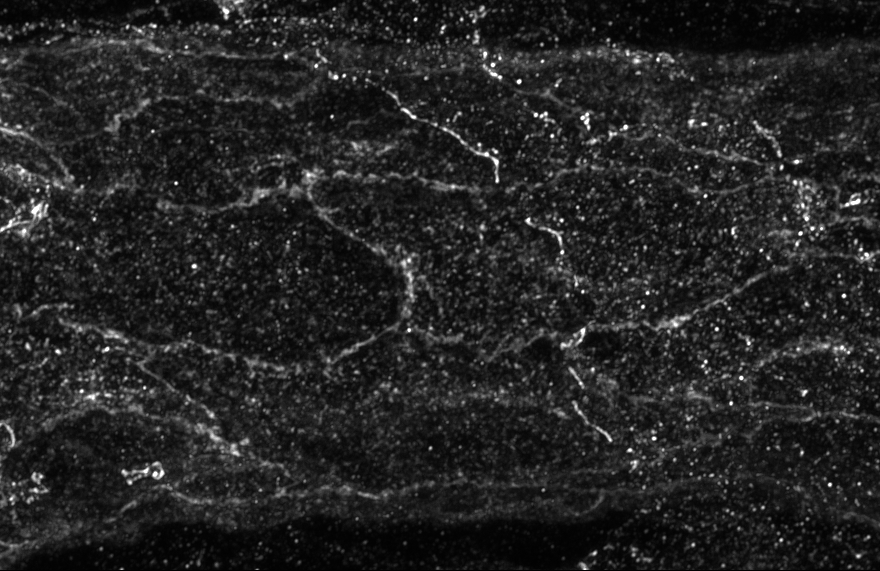

Supplement: Supplementary file 8 — Source data Fig. 7 [file 44319_2024_221_MOESM8_ESM.zip › EMBOR-2023-58528V1_SourceDataForFigure7/7B/K626_633R_VE_cadherin.tif]

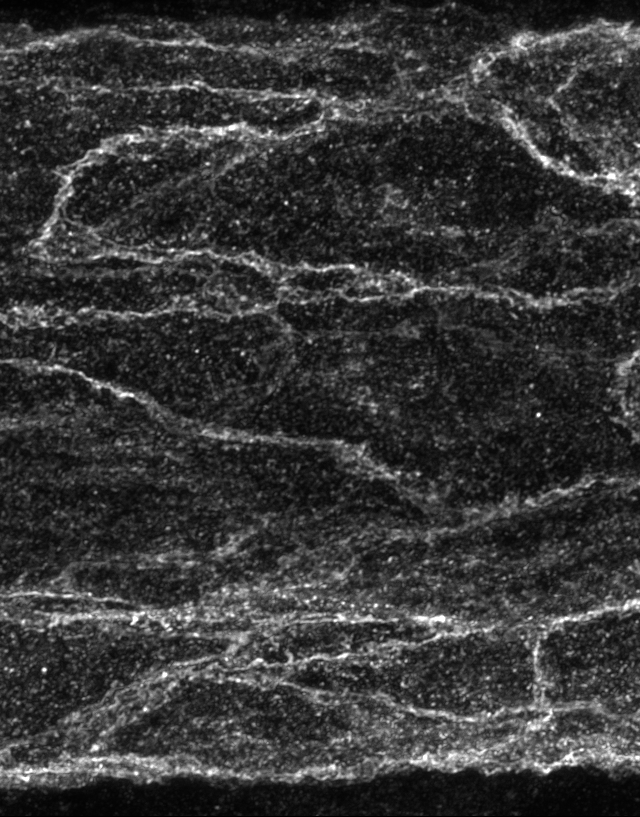

Supplement: Supplementary file 8 — Source data Fig. 7 [file 44319_2024_221_MOESM8_ESM.zip › EMBOR-2023-58528V1_SourceDataForFigure7/7B/KallR_VE_cadherin.tif]

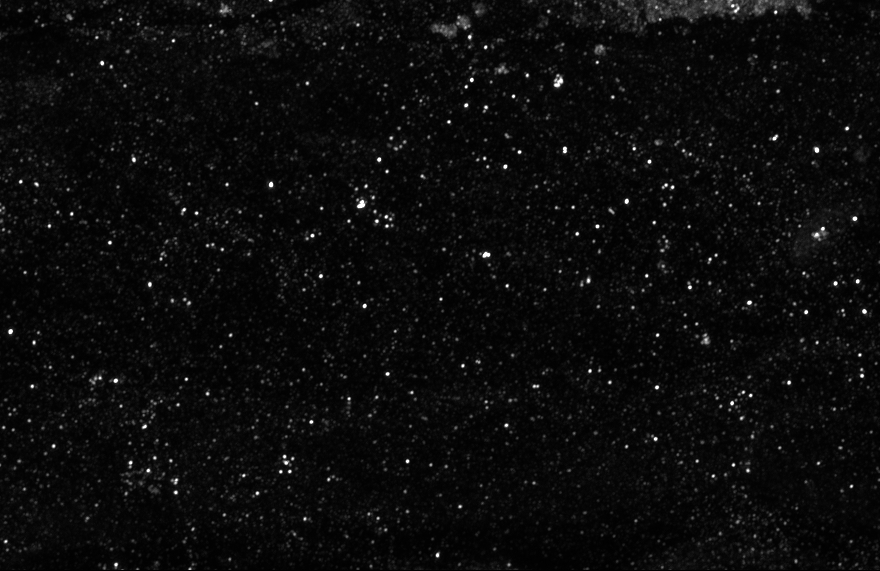

Supplement: Supplementary file 8 — Source data Fig. 7 [file 44319_2024_221_MOESM8_ESM.zip › EMBOR-2023-58528V1_SourceDataForFigure7/7B/K626_633R_MVB.tif]

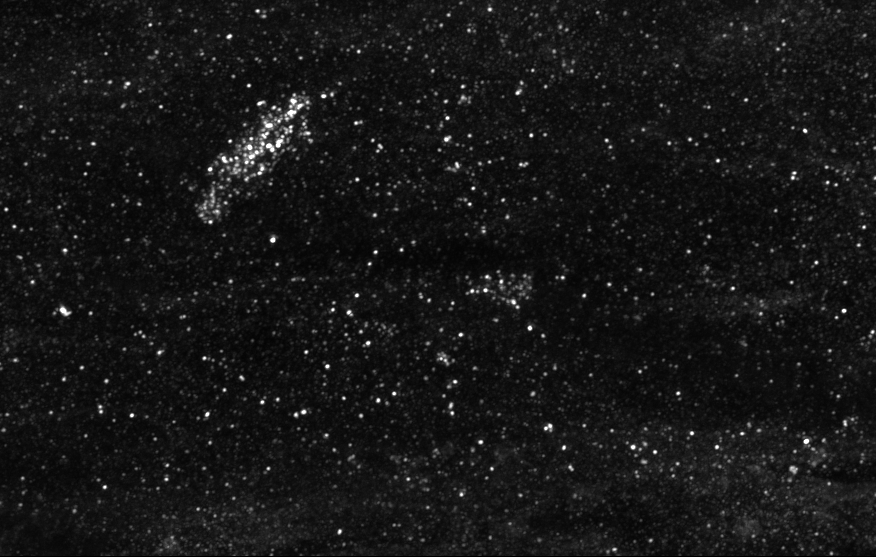

Supplement: Supplementary file 8 — Source data Fig. 7 [file 44319_2024_221_MOESM8_ESM.zip › EMBOR-2023-58528V1_SourceDataForFigure7/7B/WT_MVB.tif]

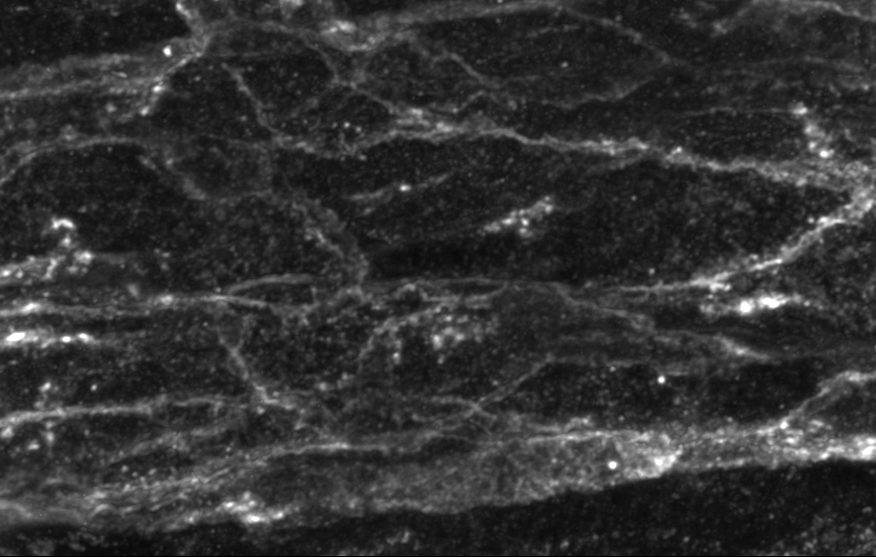

Supplement: Supplementary file 8 — Source data Fig. 7 [file 44319_2024_221_MOESM8_ESM.zip › EMBOR-2023-58528V1_SourceDataForFigure7/7B/WT_VE_cadherin.tif]

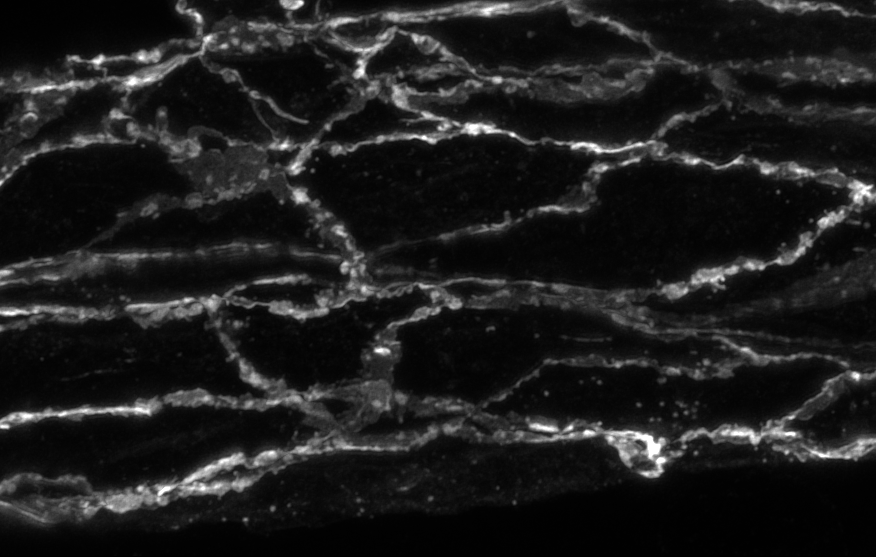

Supplement: Supplementary file 8 — Source data Fig. 7 [file 44319_2024_221_MOESM8_ESM.zip › EMBOR-2023-58528V1_SourceDataForFigure7/7B/WT_PECAM.tif]

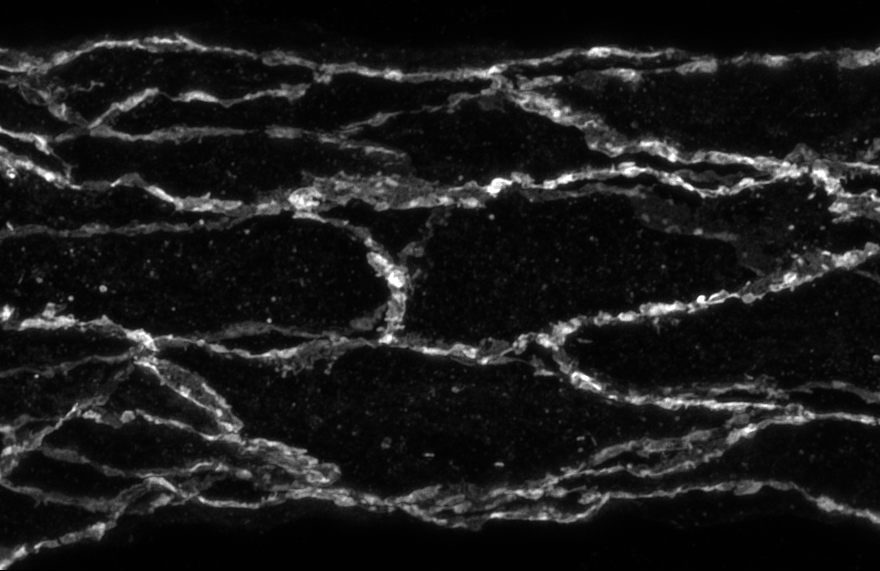

Supplement: Supplementary file 8 — Source data Fig. 7 [file 44319_2024_221_MOESM8_ESM.zip › EMBOR-2023-58528V1_SourceDataForFigure7/7B/K626_633R_PECAM.tif]

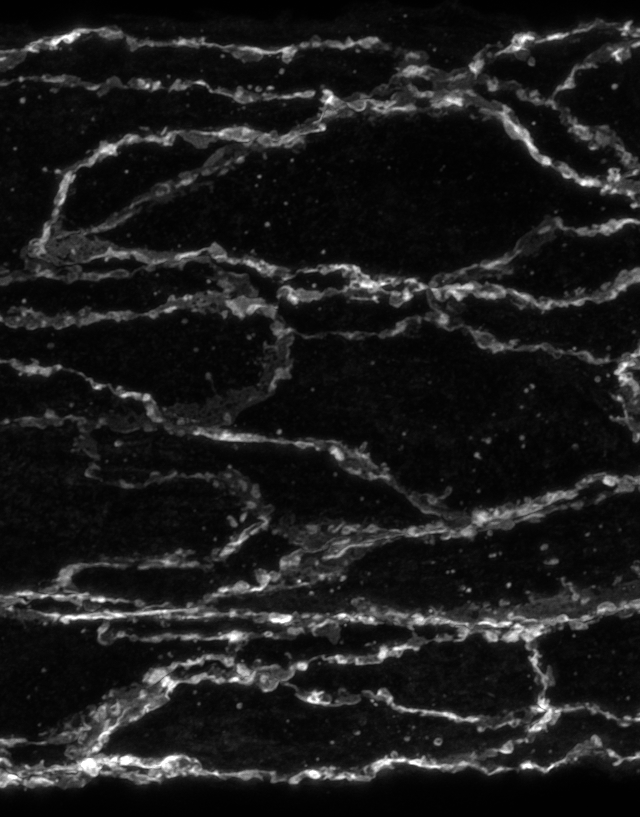

Supplement: Supplementary file 8 — Source data Fig. 7 [file 44319_2024_221_MOESM8_ESM.zip › EMBOR-2023-58528V1_SourceDataForFigure7/7B/KallR_PECAM.tif]

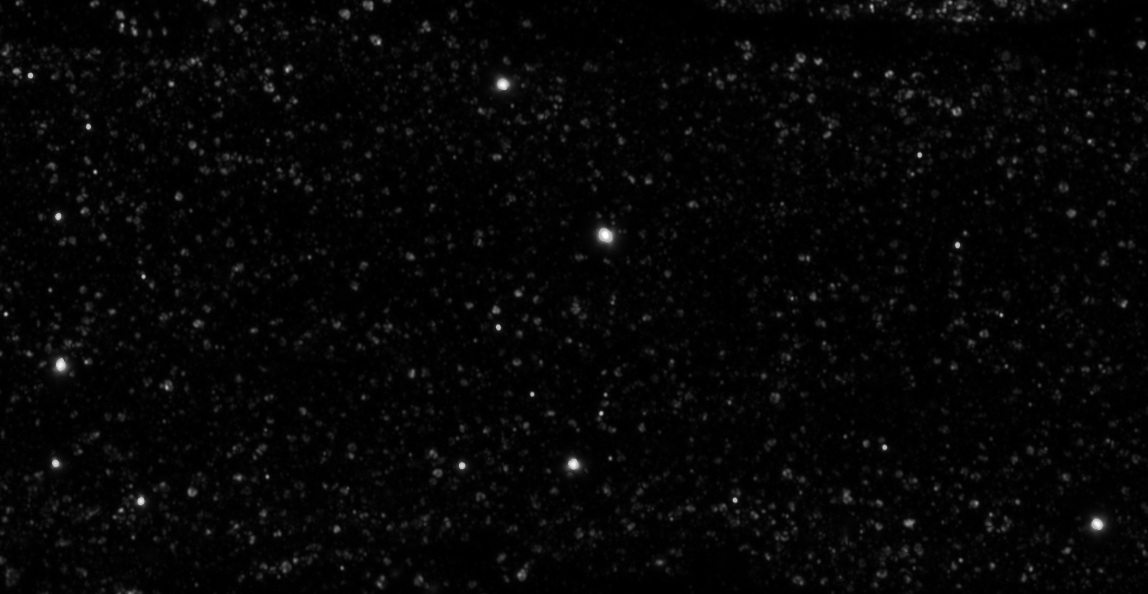

Supplement: Supplementary file 8 — Source data Fig. 7 [file 44319_2024_221_MOESM8_ESM.zip › EMBOR-2023-58528V1_SourceDataForFigure7/7A/KallR_EE.tif]

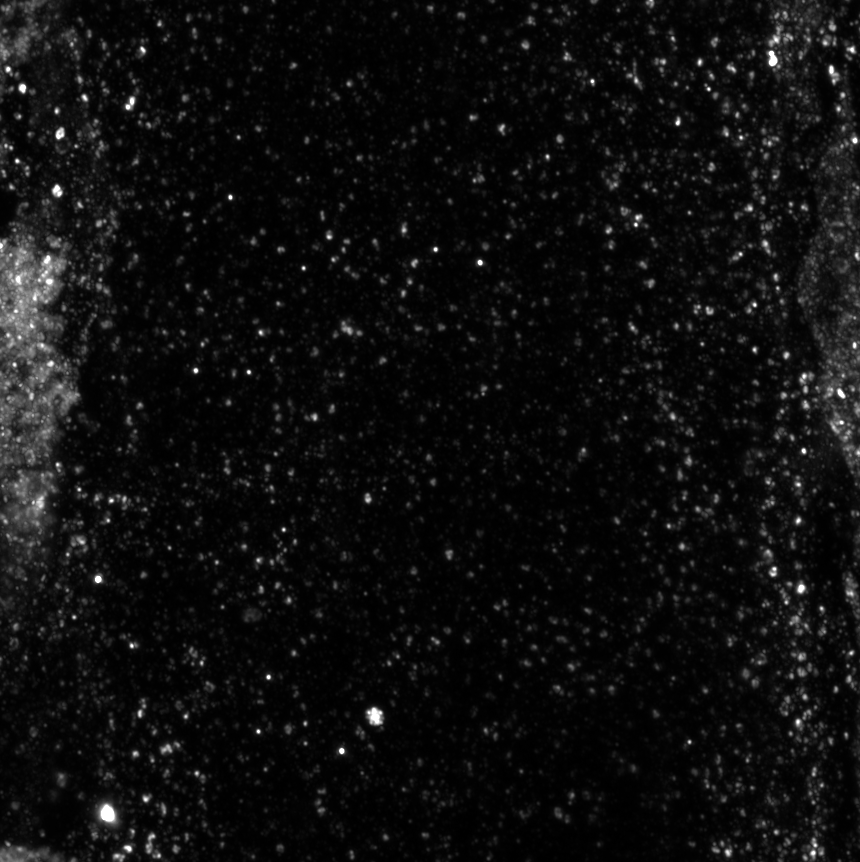

Supplement: Supplementary file 8 — Source data Fig. 7 [file 44319_2024_221_MOESM8_ESM.zip › EMBOR-2023-58528V1_SourceDataForFigure7/7A/K626_633R_EE.tif]

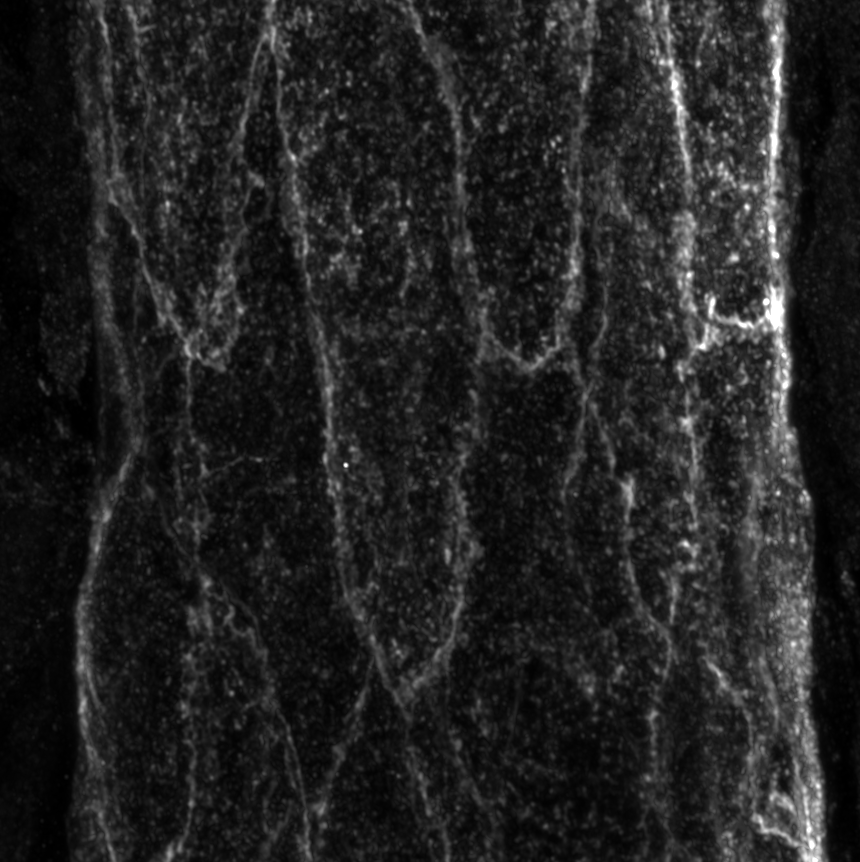

Supplement: Supplementary file 8 — Source data Fig. 7 [file 44319_2024_221_MOESM8_ESM.zip › EMBOR-2023-58528V1_SourceDataForFigure7/7A/K626_633R_VE_cadherin.tif]

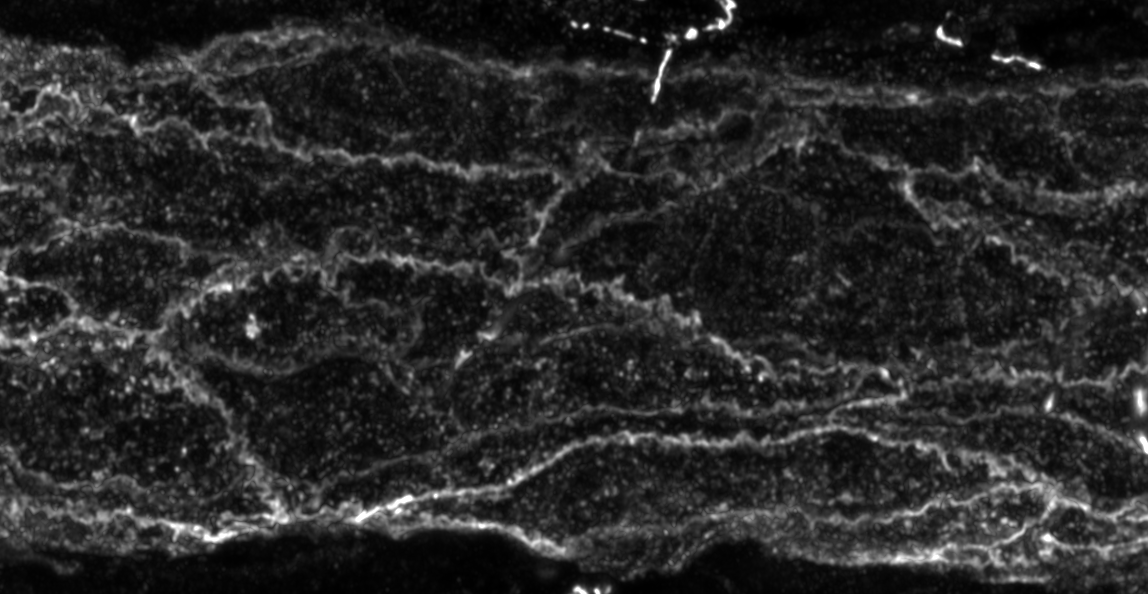

Supplement: Supplementary file 8 — Source data Fig. 7 [file 44319_2024_221_MOESM8_ESM.zip › EMBOR-2023-58528V1_SourceDataForFigure7/7A/KallR_VE_cadherin.tif]

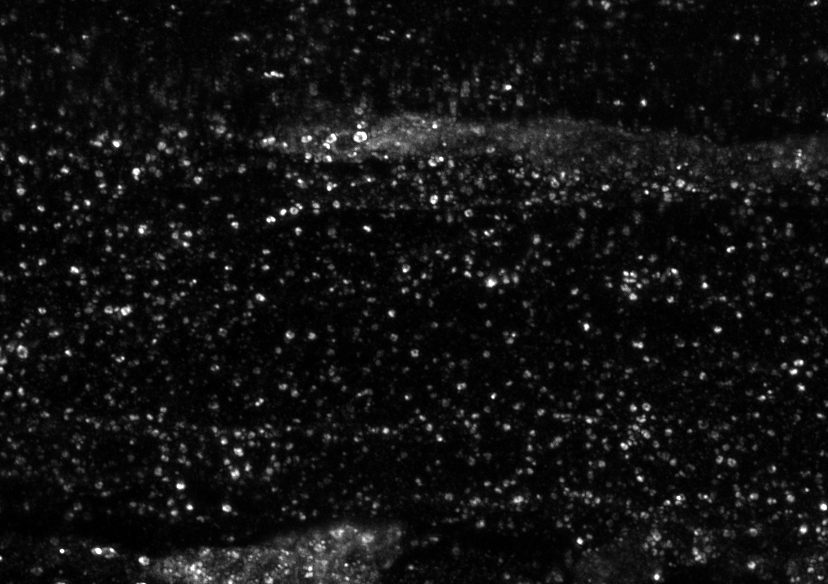

Supplement: Supplementary file 8 — Source data Fig. 7 [file 44319_2024_221_MOESM8_ESM.zip › EMBOR-2023-58528V1_SourceDataForFigure7/7A/WT_EE.tif]

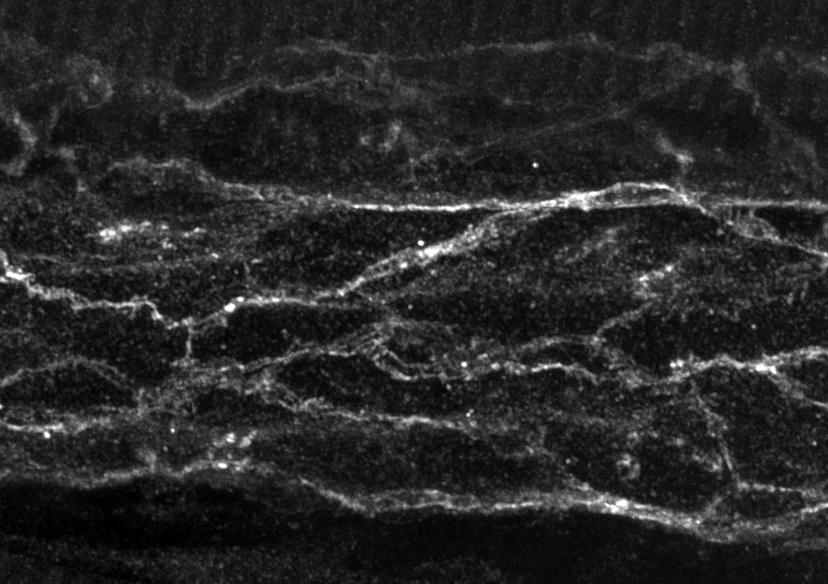

Supplement: Supplementary file 8 — Source data Fig. 7 [file 44319_2024_221_MOESM8_ESM.zip › EMBOR-2023-58528V1_SourceDataForFigure7/7A/WT_VE_cadherin.tif]

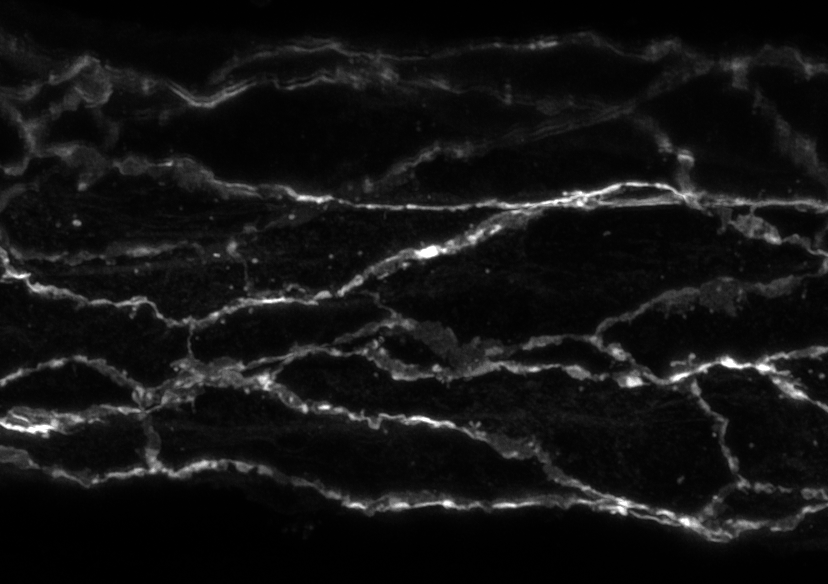

Supplement: Supplementary file 8 — Source data Fig. 7 [file 44319_2024_221_MOESM8_ESM.zip › EMBOR-2023-58528V1_SourceDataForFigure7/7A/WT_PECAM.tif]

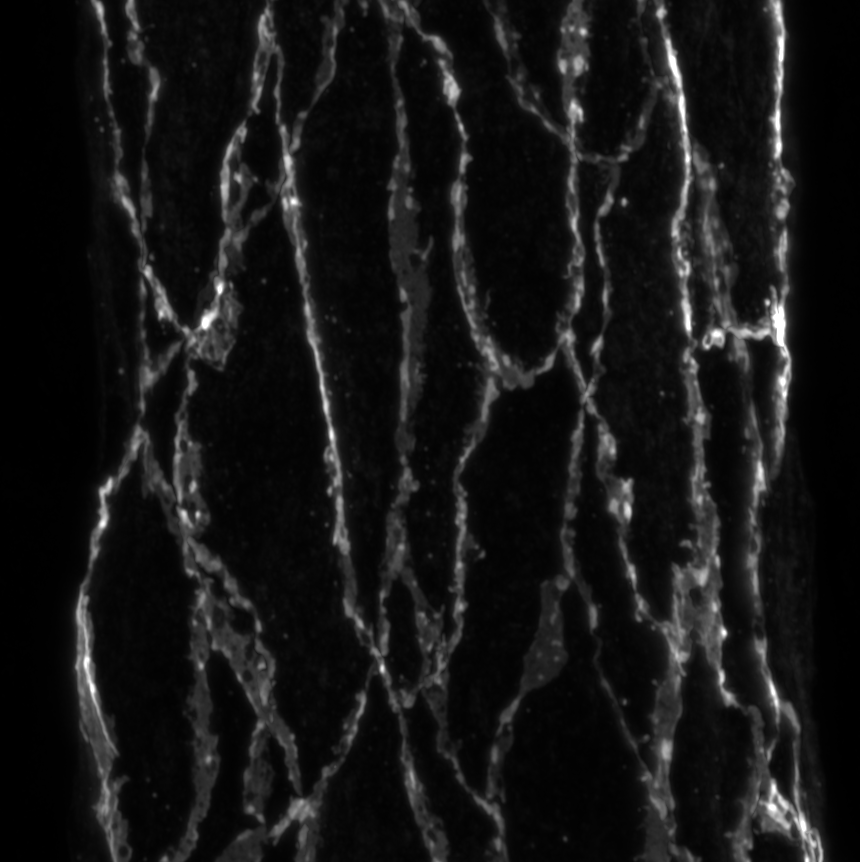

Supplement: Supplementary file 8 — Source data Fig. 7 [file 44319_2024_221_MOESM8_ESM.zip › EMBOR-2023-58528V1_SourceDataForFigure7/7A/K626_633R_PECAM.tif]

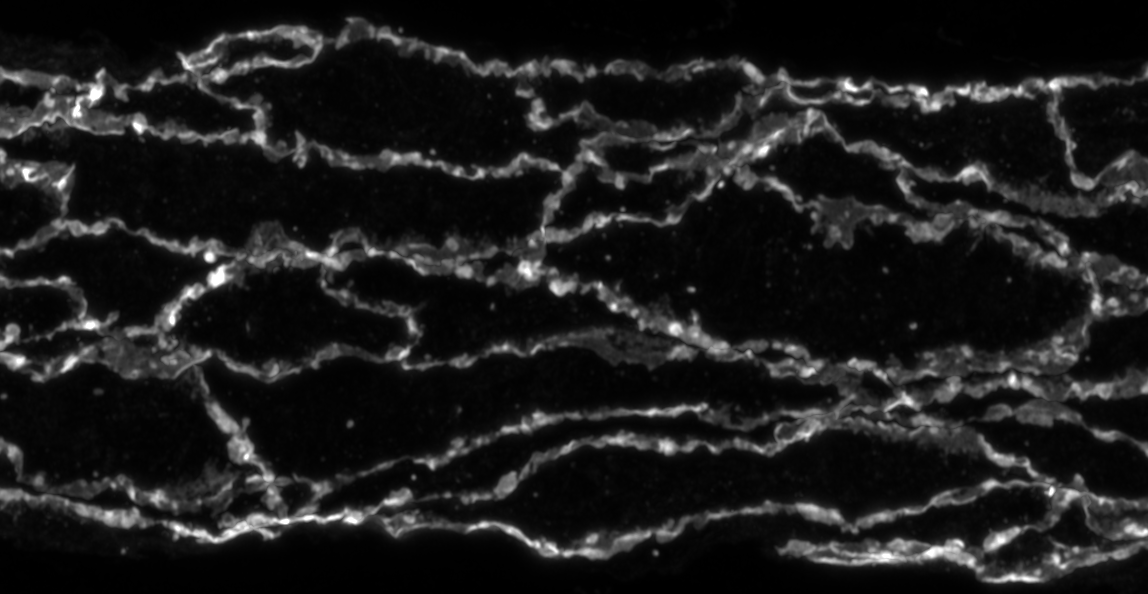

Supplement: Supplementary file 8 — Source data Fig. 7 [file 44319_2024_221_MOESM8_ESM.zip › EMBOR-2023-58528V1_SourceDataForFigure7/7A/KallR_PECAM.tif]

# Figure 8A Blots

A

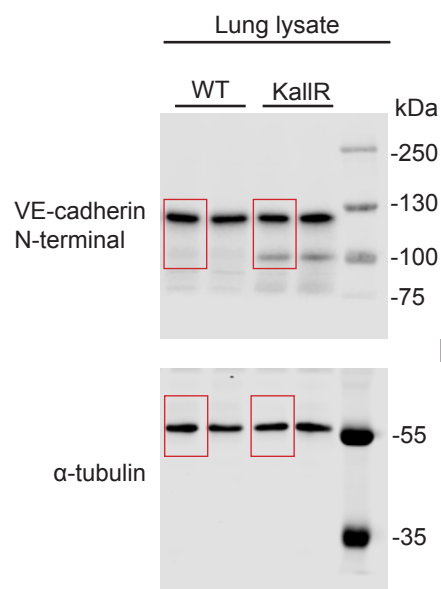

Blot was cut in half at 75 kDa

Supplement: Supplementary file 9 — Source data Fig. 8 [file 44319_2024_221_MOESM9_ESM.zip › EMBOR-2023-58528V1_SourceDataForFigure8/8A/Figure_8A_Blots.pdf]

# Figure 8D Blots

D

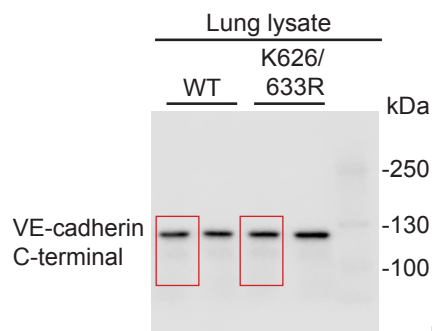

Blot was cut in half at 75 kDa

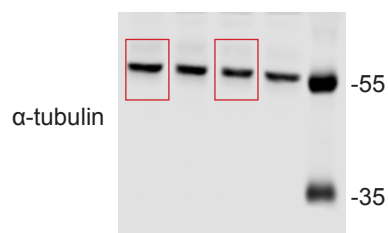

Supplement: Supplementary file 9 — Source data Fig. 8 [file 44319_2024_221_MOESM9_ESM.zip › EMBOR-2023-58528V1_SourceDataForFigure8/8D/Figure_8D_Blots.pdf]

# Figure 8C Blots

C

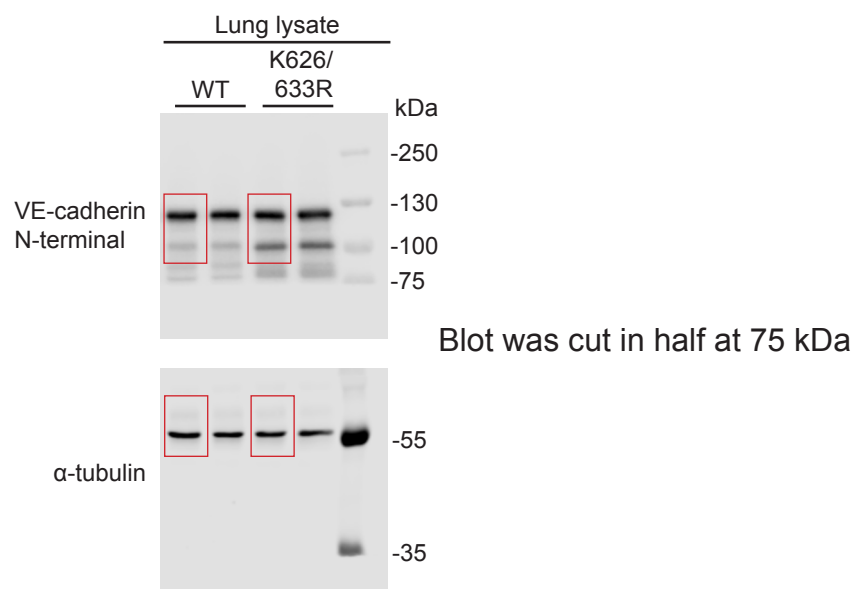

Supplement: Supplementary file 9 — Source data Fig. 8 [file 44319_2024_221_MOESM9_ESM.zip › EMBOR-2023-58528V1_SourceDataForFigure8/8C/Figure_8C_Blots.pdf]

# Figure 8B Blots

B

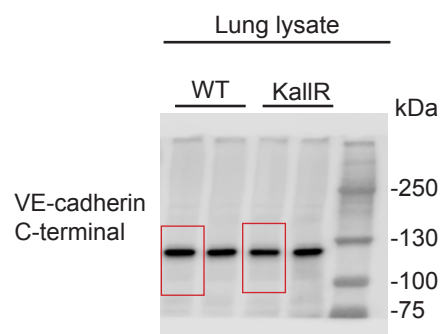

Blot was cut in half at 75 kDa

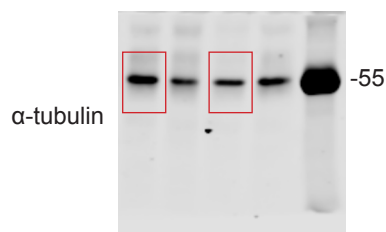

Supplement: Supplementary file 9 — Source data Fig. 8 [file 44319_2024_221_MOESM9_ESM.zip › EMBOR-2023-58528V1_SourceDataForFigure8/8B/Figure_8B_Blots.pdf]
