# Supplementary material for: Ubiquitination of VE-cadherin regulates inflammation-induced vascular permeability in vivo
Source: EMBO Rep. 2024 Aug 7;25(9):17. doi: 10.1038/s44319-024-00221-7 (PMC11387630; doi:10.1038/s44319-024-00221-7)
Supplement: Supplementary file 3 — Source data Fig. 2 [file 44319_2024_221_MOESM3_ESM.zip › EMBOR-2023-58528V1_SourceDataForFigure2/2C/Biotinylation_Assay_SourceData.pdf]

Figure 2 A

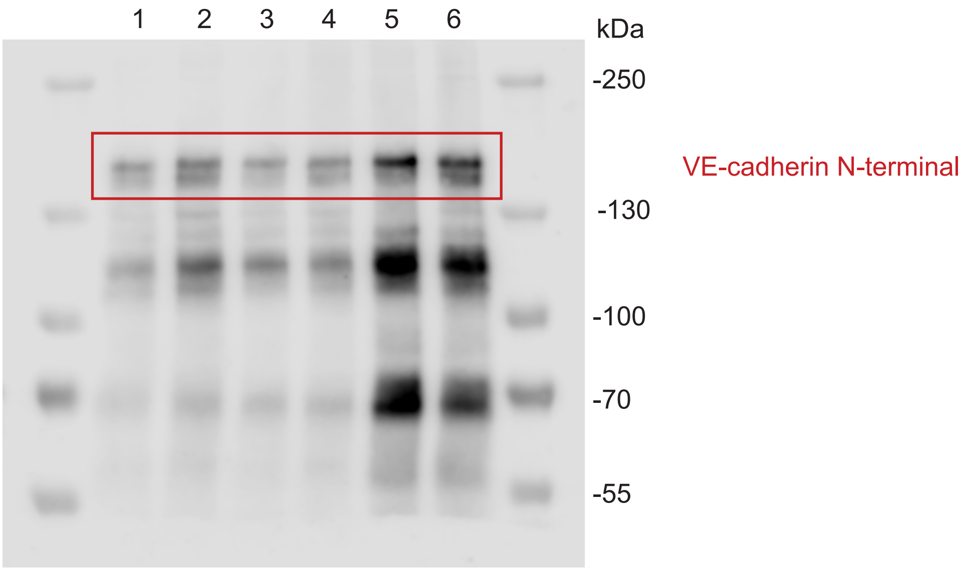

1: VEC-WT Control
2: VEC-WT + 10 min histamine
3: VEC-Y685F Control
4: VEC-Y685F + 10 min histamine
5: VEC-K626/633R Control
6: VEC-K626/633R + 10 min histamine
