# Supplementary material for: Ubiquitination of VE-cadherin regulates inflammation-induced vascular permeability in vivo
Source: EMBO Rep. 2024 Aug 7;25(9):17. doi: 10.1038/s44319-024-00221-7 (PMC11387630; doi:10.1038/s44319-024-00221-7)
Supplement: Supplementary file 9 — Source data Fig. 8 [file 44319_2024_221_MOESM9_ESM.zip › EMBOR-2023-58528V1_SourceDataForFigure8/8G/Bafilomycin_SourceData.pdf]

Figure 8 A

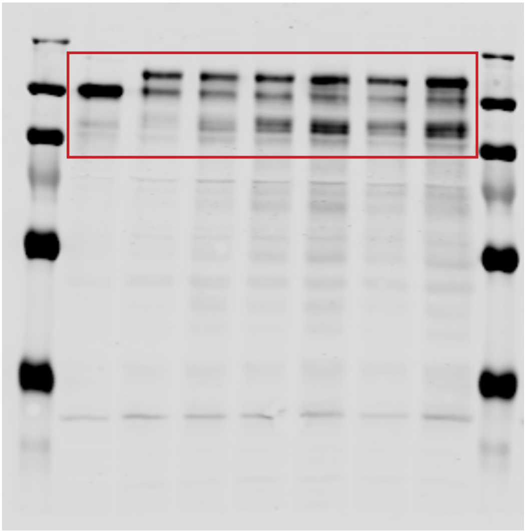

1: Control siRNA
2: VEC-WT Control
3: VEC-WT + 6h bafilomycin
4: VEC-K626/633R Control
5: VEC-K626/633R + 6h bafilomycin
6: VEC-K*all*R Control
7: VEC-K*all*R + 6h bafilomycin

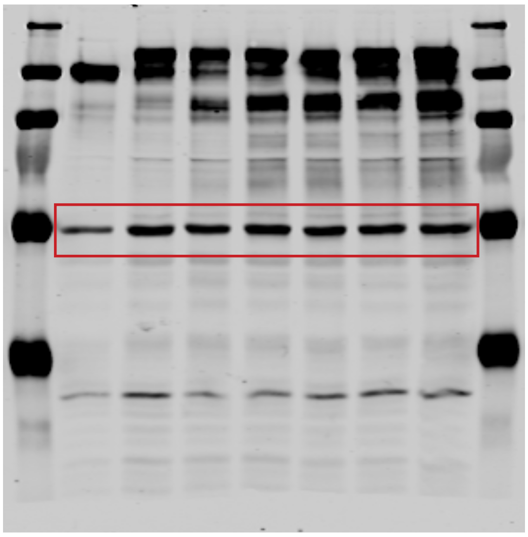

1: Control siRNA
2: VEC-WT Control
3: VEC-WT + 6h bafilomycin
4: VEC-K626/633R Control
5: VEC-K626/633R + 6h bafilomycin
6: VEC-K*all*R Control
7: VEC-K*all*R + 6h bafilomycin
