# Supplementary material for: Ubiquitination of VE-cadherin regulates inflammation-induced vascular permeability in vivo
Source: EMBO Rep. 2024 Aug 7;25(9):17. doi: 10.1038/s44319-024-00221-7 (PMC11387630; doi:10.1038/s44319-024-00221-7)
Supplement: Supplementary file 10 — Expanded View Figures [file 44319_2024_221_MOESM10_ESM.pdf]

Expanded View Figures

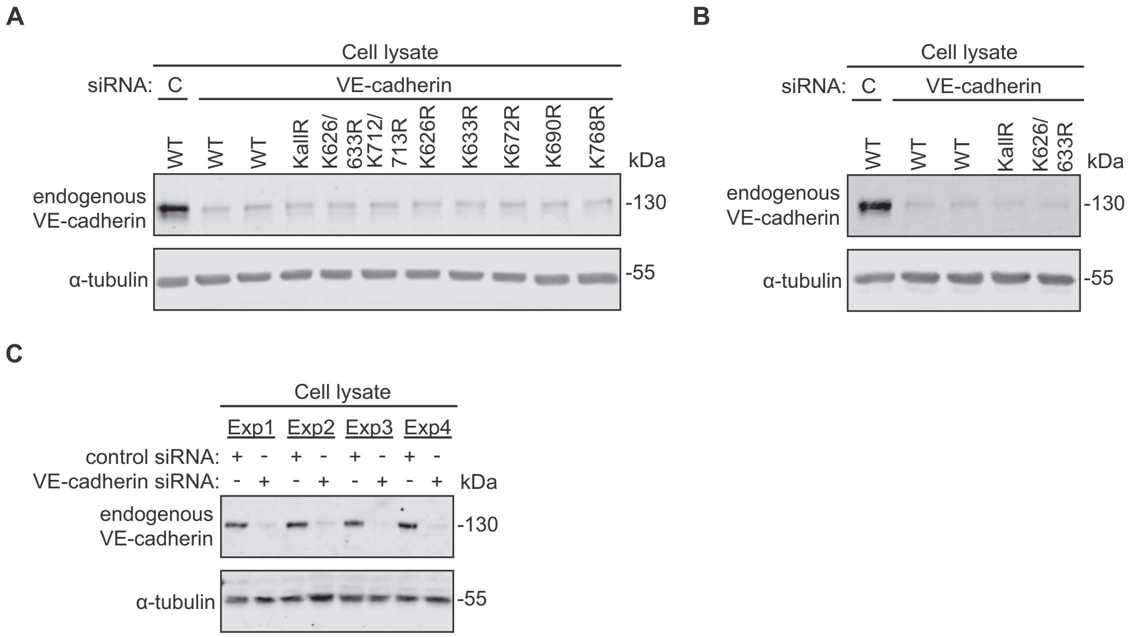

Figure EV1. Endogenous VE-cadherin knockdown efficiency for experiments in Fig. 1.

(**A–C**) Endogenous VE-cadherin was silenced in HUVEC via VE-cadherin siRNA. Whole cell lysates were immunoblotted for endogenous VE-cadherin and α-tubulin. Molecular sizes are indicated in kilodaltons (kDa). (**A**) Experiment shown in Fig. 1B. (**B**) Experiment shown in Fig. 1D. (**C**) Experiment shown in Fig. 1F.

 © The Author(s)

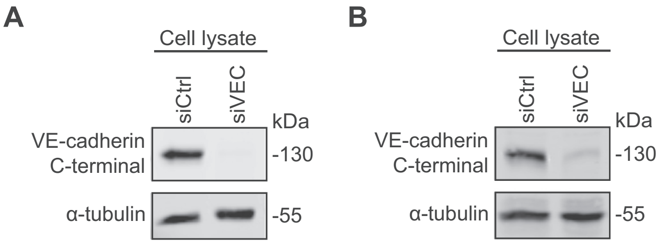

Figure EV2. Endogenous VE-cadherin knockdown efficiency for experiments in Fig. 2.

(**A**, **B**) Endogenous VE-cadherin was silenced in HUVEC via VE-cadherin siRNA. Whole cell lysates were immunoblotted for endogenous VE-cadherin and α-tubulin. Molecular sizes are indicated in kilodaltons (kDa). (**A**) Experiment shown in Fig. 2A. (**B**) Experiment shown in Fig. 2C.

© The Author(s)

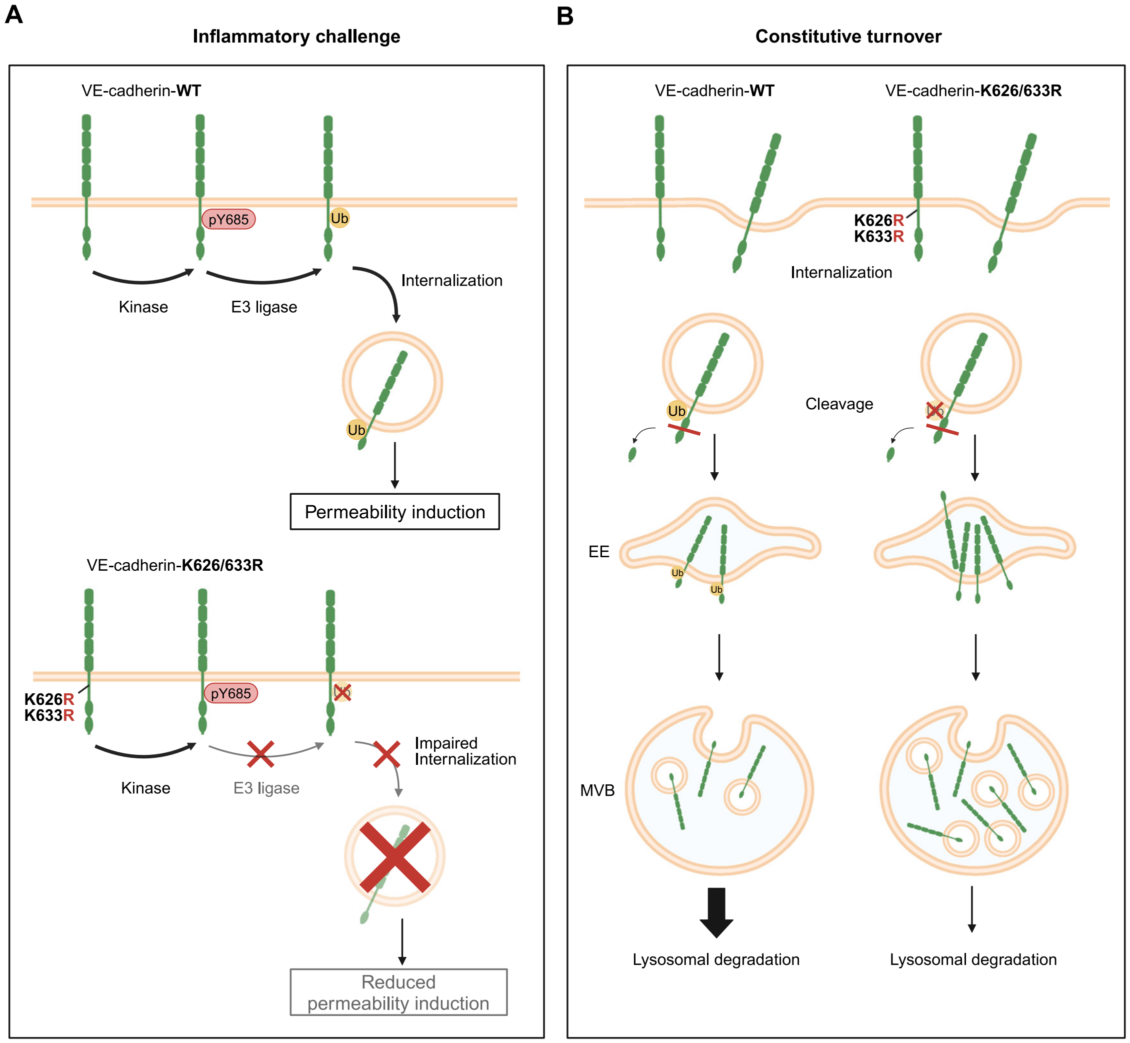

Figure EV3. Dual function of VE-cadherin ubiquitination in inflammation-induced endocytosis and lysosomal targeting.

(**A**) Upon inflammatory challenge, Y685 of VE-cadherin gets phosphorylated followed by ubiquitination of VE-cadherin lysine residues K626 and K633, leading to VE-cadherin endocytosis and an increase in vascular permeability. This is prevented when K626 and K633 are mutated to arginine leading to impaired induction of vascular permeability. (**B**) Besides inflammation-induced endocytosis, ubiquitination of VE-cadherin controls a second process, the targeting of VE-cadherin to the lysosomal degradation pathway. Mutation of K626 and K633 to arginine slows down lysosomal targeting of mutated VE-cadherin leading to its accumulation in early endosomes (EE) and multivesicular bodies (MVB). Our results imply that K/R mutated VE-cadherin can be constitutively endocytosed in resting cells by lysine independent mechanisms, and therefore accumulates inside the cell, due to impaired degradation.

 © The Author(s)
